# Corticosterone-implanted chicks transmit stress to parents and neighbors in a colonial seabird

**DOI:** 10.1093/beheco/araf085

**Published:** 2025-07-28

**Authors:** Susana Cortés-Manzaneque, Sin-Yeon Kim, Alberto Velando

**Affiliations:** Grupo Ecoloxía Animal, Centro de Investigación Mariña, Universidade de Vigo, 36310 Vigo, Spain; Grupo Ecoloxía Animal, Centro de Investigación Mariña, Universidade de Vigo, 36310 Vigo, Spain; Grupo Ecoloxía Animal, Centro de Investigación Mariña, Universidade de Vigo, 36310 Vigo, Spain

**Keywords:** corticosterone, phenotypic programming, social transmission, stress crossover, stress response

## Abstract

In animals living in groups, stress-induced changes in behavior can be a source of social information, and stressed individuals can potentially become stressors for other social partners, with important consequences for social and population dynamics. Here, we studied stress transmission from experimentally stressed chicks to both their parents and neighbors in the yellow-legged gull (*Larus michahellis*), a seabird that forms large breeding colonies. To do this, we experimentally increased the level of a stress hormone by corticosterone implant in 2 first-hatched chicks of the brood and observed its effects on their parents and both adults and chicks in the neighboring nests. Two days after the implant, corticosterone-implanted chicks showed reduced basal corticosterone levels, probably due to a physiological feedback response. Exogenous corticosterone promoted behavioral changes in the corticosterone-implanted chicks, showing faster responses to a potential predator attack than the placebo-treated chicks. Eight days after implantation, not only the corticosterone-implanted chicks but also the neighboring chicks showed elevated corticosterone levels after a standardized handling stress compared with the placebo-implanted chicks and their neighbors. The parents and neighbor adults of the corticosterone-implanted chicks showed increased mobbing behavior but reduced aggressive and resting behaviors in comparison with the adult gulls living close to the placebo-implanted chicks. Overall, our results suggest that individual physiological stress in a colony may be socially transmitted within families and neighbors, with potential consequences for colony dynamics.

## Teaser

Animals rely on stress responses to cope with environmental challenges, but can they also perceive and react to the stress experienced by their neighbors? In our study on a colonial seabird, experimental stress was transmitted from chicks to their parents and neighbors and changed their behaviors. Our results show that socially transmitted stress shapes how animal groups respond to environmental threats.

## Introduction

Group-living animals often use social information from the behavior of conspecifics to adjust their own behavior (Dall et al. 2005; Morand-Ferron et al. 2010; Wagner and Danchin 2010). The use of social information is thought to allow individuals to respond quickly and effectively to prevailing environmental conditions (Danchin et al. 2004; Bonnie and Earley 2007; Weimerskirch et al. 2010). In vertebrates, environmental threats commonly activate the hypothalamic–pituitary–adrenal axis, leading to the release of glucocorticoids, which induce changes in individual behaviors across different contexts (Romero 2002; Myers et al. 2014). Recent evidence suggests that individuals with elevated glucocorticoids may transmit stress socially to other individuals in close proximity (eg brood mates in a natural population: Noguera et al. 2017; neighbors in captivity: Brandl and Farine 2024). Social contagion of stress responses may have important consequences for collective behavior and the dynamics of populations (Jovani and Grimm 2008; Creel et al. 2013; Brandl et al. 2022). However, it remains unclear whether stress is transmitted beyond the brood level, such as to parents or neighbors, and how this transmission might influence the functioning of social groups in natural populations.

Breeding colonies are a remarkable form of group living, characterized by high densities and close contact of conspecifics found in a wide range of taxa, including colonial birds and mammals (Danchin and Wagner 1997). It has been proposed that social information may be one of the principal advantages of coloniality, favoring, eg rapid and coordinated responses to predators (Arroyo et al. 2001; Jungwirth et al. 2015; Evans et al. 2016). Indeed, many colonial animals show collective group defense behavior, such as the coordinated mobbing against predators of several seabird species (Clode et al. 2000; Kazama and Watanuki 2010). In this social process, developing individuals may serve as important sources of social information for their parents and other colony members. Although remaining in the natal territory and interacting with neighbors (Charrier et al. 2001; Ashbrook et al. 2008; Salas et al. 2024), young animals are continuously exposed to social cues and probably relay information about local stressors to their parents or other colony members. Under high predation risk, social transmission of stress may prepare nearby colony members to collectively respond to predators, although few individuals interact directly with predators during the collective defense (Boonstra 2013). In contrast, in the absence of predators, nonstressed individuals adaptively reduce their vigilance time and spend more time on other activities, such as grooming, resting, and caring for young, thereby increasing their fitness (Elgar 1989; Picman et al. 2002).

In vertebrates, the hypothalamic–pituitary–adrenal axis integrates environmental threats and internal states, transmitting this information to body organs through blood glucocorticoids, leading to physiological and behavioral responses that help the animal to cope with stressful situations (reviewed in Boonstra 2005; Romero and Butler 2007). For example, the exposure to predators triggers a rapid response of the hypothalamic–pituitary–adrenal axis, resulting in a spike in glucocorticoids, which induces immediate adaptive responses, such as antipredator behavior for survival (Sapolsky et al. 2000; Wingfield and Romero 2011). Experimental increases in circulating glucocorticoid levels in developing animals alter their behavior, showing stress-related behavioral phenotypes (Kitaysky et al. 2003; Schoech et al. 2011; Marasco et al. 2012). Thus, the experimental manipulation of corticosterone and response behavior may allow us to test whether stressed young transmit their stress to other social members within the colony.

Here, we performed such an experiment and investigated whether the exposure to corticosterone implants affects stress-related behavioral phenotypes in the yellow-legged gull (*Larus michahellis*) chicks and whether these changes affect the behavior of their parents and other conspecifics in neighboring nests. Colonial gulls are ideal models for studying stress contagion, as they congregate to breed in small and dense territories and display high levels of social interactions between neighboring nests, including communal defense and agonistic interactions (Petterolf 1984). Gulls are known to socially transmit relevant ecological information, such as the location of food and the presence of predators, to other colony members (Evans 1982; Beauchamp 2009; Monier 2024). In gull colonies, information exchange is probably responsible for synchronized breeding and foraging at the level of subcolonies or small neighborhoods (Burger 1979; Evans and Welham 1985; Immer et al. 2021). In the yellow-legged gull, females transfer information about the presence of predators to their offspring via egg substances (Morales et al. 2018), such as corticosterone (Rubolini et al. 2005), and developing embryos respond to alarm calls emitted by adults and transmit this information to brood-mate embryos, probably by vibration (Noguera and Velando 2019). A previous study showed that corticosterone implants alter the behavior of chicks, and these changes trigger stress responses in brood mates, thereby providing evidence for stress transmission within the brood (Noguera et al. 2017). In this study, we examined the effects of corticosterone-implanted chicks on the behavior of their parents and neighbor adults.

For this experiment, we selected pairs of 2 nearby nests with the modal clutch size of 3 eggs (hereafter nest dyads). At hatching, 2 core chicks in 1 nest of each nest dyad were implanted with either corticosterone or placebo, whereas the neighbor chicks were not manipulated. Thus, in the corticosterone dyads, 1 brood had elevated levels of exogenous corticosterone, whereas in the placebo dyads, 1 brood was manipulated similarly but without corticosterone exposure. First, we evaluated whether exogenous corticosterone immediately affected stress-related behaviors in the implanted chicks, as this was the main assumption underlying our manipulation. We expected that the corticosterone-implanted chicks would hide more quickly in response to threats and show reduced begging behavior compared with the placebo-implanted chicks (Romero and Butler 2007; Noguera et al. 2017). This experimental change of chick behavior should allow us to test whether the stress response of the corticosterone-implanted chicks is transferred to their social environment.

Second, to test for the transmission of stress to the neighboring chicks, we examined their physiological stress responses and growth throughout their development. Eight days after implantation, when the exogenous corticosterone had been released and chicks had active social interactions with their neighbors, we captured both the manipulated and unmanipulated chicks to examine basal corticosterone level, body size, and adrenocortical response to a standardized stress protocol (capture followed by 30 min restraint). We also captured fully grown chicks near fledging to examine basal corticosterone level and body size. Given the frequent social interactions between neighboring families in colonial gulls, we expected that both the corticosterone-implanted chicks and their neighbor chicks would increase physiological stress responses, thereby experiencing long-lasting negative consequences for growth (Noguera et al. 2017).

Third, in parallel, we studied the effect of cross-stress on the behavior of adults within their breeding territory and neighborhood while corticosterone implants were active. Because social transmission of stress may influence the collective behavior of animal groups (Bonnie and Earley 2007; Brandl and Farine 2024), we predicted that the parents and neighbor adults of the corticosterone-implanted chicks would show more defensive behaviors, such as increased mobbing and reduced resting, as they would remain more vigilant to predation risks.

## Materials and methods

### Study system

The yellow-legged gull is a monogamous seabird that breeds in dense colonies where adults engage in communal defense against predators through mobbing and maintain territorial boundaries by aggressive encounters with neighbors throughout the breeding season (Tinbergen 1953). Within each territory, breeding pairs construct small nests and typically lay a clutch of 3 eggs. In our study population, the first 2 chicks usually hatch synchronously and show comparable competitive abilities, whereas the third chick, which hatches later, suffers a competitive disadvantage (Kim et al. 2011; Díaz-Real et al. 2016). Gull chicks are semiprecocial, remaining in the nest for the first 2 d, after which they begin to explore their territory and engage in social interactions with both their parents (Morales et al. 2009) and nearby conspecifics (Beer 1979; Salas et al. 2024).

In gulls, both parents provide care, and feeding is typically initiated by parental calls upon landing, which elicit conspicuous begging behavior from the chicks to attract parental attention (Tinbergen and Perdeck 1950; Impekoven 1971; Rubolini et al. 2005; Noguera et al. 2013). The intensity of begging is related to chick body mass, which reflects both nutritional condition and maternal investment via egg quality (Pérez et al. 2006; Noguera et al. 2010; Kim et al. 2011). Gull chicks do not recognize their siblings or parents until 4 to 5 d post hatching (Evans 1970). This species displays sexual size dimorphism, and sex-related differences have been documented in chick behavior (Kim et al. 2011; Morales et al. 2018), physiology (Kim et al. 2013; Velando et al. 2019), and gene expression (Díaz-Real et al. 2016).

### Study area and general procedures

We performed the field experiment between April and July 2022 in a large breeding colony of yellow-legged gulls situated on Sálvora Island, part of the Parque Nacional das Illas Atlánticas de Galicia (42°28′N, 09°00′W), Spain. We surveyed dense breeding areas daily to mark nests, record egg laying, and measure egg length and width, for calculating egg volume (Hoyt 1979). In total, we monitored 206 nests of known laying dates and order. Among these monitored clutches, after clutch completion, we selected 40 pairs of nearby nests (nest dyads; 80 nests in total) with 3 eggs (modal clutch size) that had similar laying dates within each nest dyad. Thus, each nest dyad consisted of 2 nests with similar laying dates (±1 d) that were in very close proximity to each other, facilitating social interactions among neighboring chicks and adults (average distance 4.8 m, range 1 to 11 m; see Fig. S1). To disrupt any potential stress covariation between parental and offspring phenotype and between nearby broods, we cross-fostered the whole clutch between focal nests and distant donor nests (from the monitored nest) with similar laying dates.

**Fig. 1.**
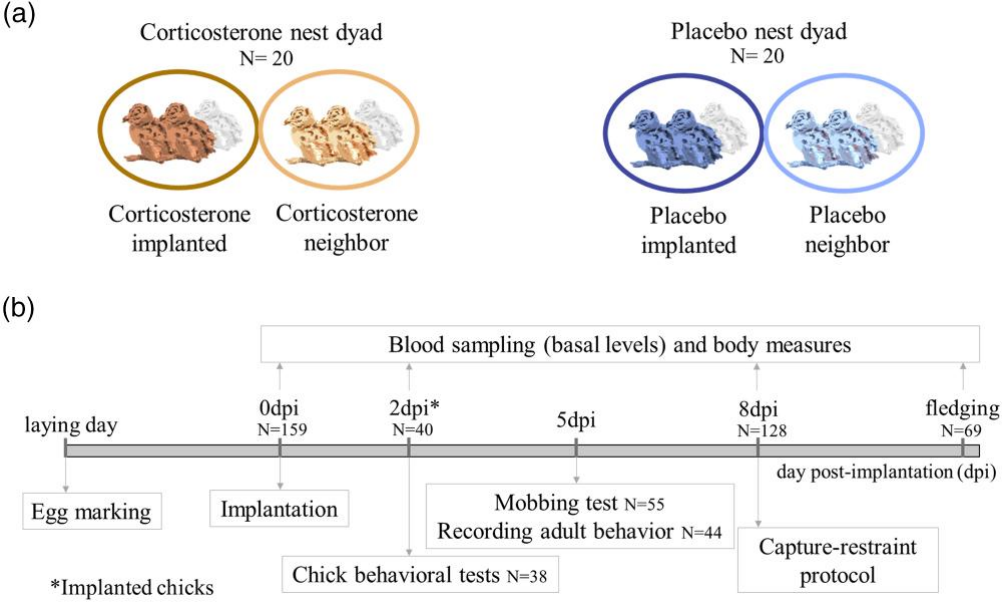
Schematic representation of the experimental design and schedule. a) Assignment of experimental chicks: corticosterone implanted, corticosterone neighbor, placebo implanted, and placebo neighbor. b) We marked eggs and nests during egg laying, and hatchlings were blood sampled and subject to the manipulation of corticosterone level (implantation). On 2 d post implantation, we sampled blood and tested the behavior in half of the implanted chicks. On 5 d post implantation, we performed the mobbing test and video recorded the adult behavior on the nest. On 8 d post implantation, we blood sampled the chicks and applied a capture-restraint protocol. The final blood sampling took place at fledging (when chicks were 26 to 36 d old).

**Fig. 2.**
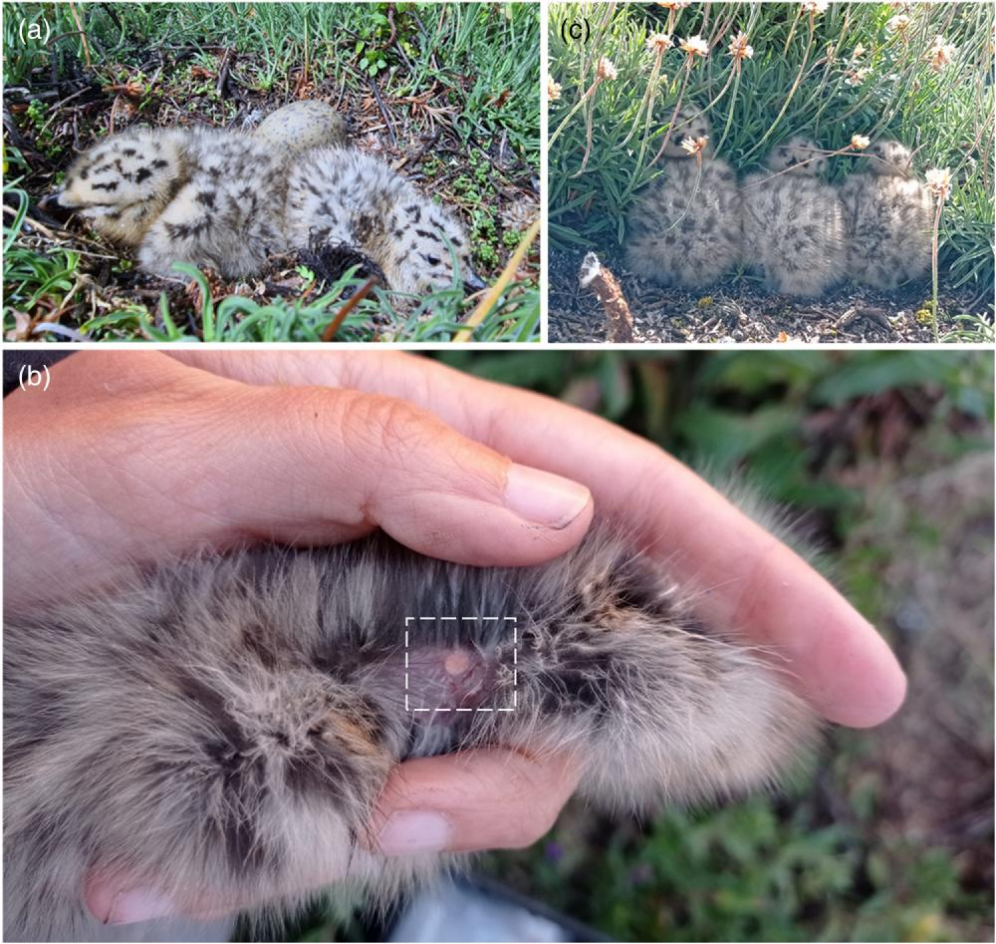
Photographs of chick behaviors on the nest and the experimental manipulation. a) First 2 chicks (*a* and *b* chicks) hatch synchronously, but the third-laid egg (*c* egg) typically hatches 2 to 3 d later. b) The pellet under the skin between the shoulders of an implanted chick. c) Chicks showing crouching behavior in response to adult alarm calls.

For this study, we used 2 first-hatched core chicks (*a* and *b* chicks) in each brood and excluded the third marginal chicks (*c* chicks), which remained undisturbed in the nests. Each nest dyad was assigned randomly to either “corticosterone” or “placebo” dyads, and 1 of the 2 broods of the nest dyads was again assigned randomly to the “implanted” treatment and the other brood to the “neighbor” (Fig. 1a). Thus, 2 chicks in 1 nest of each corticosterone dyad were implanted with corticosterone, and 2 chicks in 1 of each placebo dyad were implanted with placebo. By focusing on only the core chicks, we reduced the interference of sibling competition on stress levels (Fig. 2a). Moreover, studying synchronously hatched chicks minimized the number of visits we made per nest. We monitored each focal brood daily, beginning 2 d before the estimated hatching date until hatching of the first 2 chicks. One egg in our focal nests failed to hatch, resulting in a total of 159 focal hatchlings from 80 nests at the start of the study.

### Experimental manipulation of the stress level

To minimize disturbance, we implanted the pellets (see below) during a single visit for each nest dyad, once all chicks had hatched. In this visit, hatchlings (age 0 to 2 d; 107 0-d-old chicks, 48 1-d-old chicks, and 4 2-d-old chicks) were first blood sampled, measured (tarsus length, to the nearest 0.1 mm), and weighed (to the nearest gram; Fig. 1b). We collected blood samples (80 to 100 μl) from the brachial vein using a sterile needle and heparinized capillary tubes and kept them cold until storage in liquid nitrogen after separating plasma and red blood cells (a few hours after collection). In all cases, we collected blood samples within 3 min of capture to reduce any possible effect of handling on corticosterone level (Romero and Reed 2005). We then marked the chicks with a numbered Velcro leg tag for identification and subjected them to experimental manipulation of stress (implanted or neighboring; Fig. 1b, 0 d post implantation).

To implant corticosterone on manipulated chicks of the corticosterone dyads, we used time-release corticosterone pellets (TRPs), a commercially available matrix-driven delivery system (Innovative Research of America, Sarasota, Florida, United States) in which exogenous corticosterone is fused within a cholesterol matrix and delivered by the dual processes of erosion of the subcutaneous pellet and diffusion of the active product (catalog number: C-111). We implanted a single 1.5 mg corticosterone pellet, designed for 21 d release [theoretical dose: 0.47 to 1.1 µg corticosterone/(g‧d)], with the dose decreasing as the chicks grow. Although the manufacturer states that the effect of the pellet continues for 21 d, previous studies on birds show that the effect of the pellet decreases after about 7 d (Müller et al. 2009), probably due to the high metabolism of birds. The chosen dose is at the intermediate level of the literature, ranging from the lowest [0.01 to 0.53 µg corticosterone/(g‧d); eg Pravosudov 2003; Bonier et al. 2007; Goerlich 2009] to the highest [3.07 to 8.65 µg corticosterone/(g‧d), eg Bourgeon and Raclot 2006; Almasi et al. 2009; Müller et al. 2009; Stier et al. 2009].

Before the implantation, we sterilized the surgical equipment (scalpel and forceps) with 90% ethanol. We disinfected the skin of the chick with Iodopovidone and applied a local anesthetic spray (lidocaine; XylocainH Pumpspray, AstraZeneca) and massaged for 3 s to facilitate the distribution of the anesthetic in the implantation site, which was located between the shoulders. A small skin incision (3 mm) was then made with the scalpel, and the pellet was inserted with the forceps (Fig. 2b). The incision was then closed with a small amount of surgical glue (Vetbond 3M, St Paul, Minnesota, United States) and disinfected with a protective spray (micronized aluminum powder; Aluspray, Vetoquinol). Chicks from the implanted broods of the placebo dyads received identical implantation procedures, but with similarly prepared placebo pellets without corticosterone (catalog number: C-111). All incisions healed well, and no chick showed any lasting adverse effects from the surgical procedures. Chicks from neighbor broods of the corticosterone and placebo dyads were not subjected to implantation treatment; only blood samples and body measurements were taken, as in the manipulated chicks. Because semiprecocial gull chicks begin social interactions from 2 d of age (see above), we expect that social transmission of stress, if any, has occurred for most of the period during which the implants were releasing corticosterone (∼7 d).

### Immediate effects of corticosterone implants

We evaluated whether exogenous corticosterone induced stress-related behaviors in the implanted chicks, as this was a prerequisite for stress transmission. Two days after implantation, we examined the initial effects of the corticosterone treatment on corticosterone levels and behaviors only in the implanted chicks. During the anticipated period of corticosterone implant activity (see below), we tried to minimize disturbance and the associated stress caused by our sampling procedures. For this reason, we only (randomly) captured and sampled 1 chick from each brood that had been implanted (*N* = 20 for the placebo group and *N* = 20 for the corticosterone group). This halved the sampling time and reduced the time visiting the colony while securing enough sample size for testing the experimental prerequisite. Immediately after capture, we first blood-sampled chicks and then carefully transported them from their nests to the behavioral testing site in individual cloth bags.

### Chick begging and antipredator behaviors

We conducted behavioral tests in a quiet place outside the dense colony, following standardized protocols (Noguera et al. 2010; Kim and Velando 2015; Morales et al. 2018). The tests were always conducted at a similar time of day (between 13 and 15 h), and approximately an equal number of chicks from each experimental group were tested each day. For consecutive begging and antipredator behavior tests, we placed an individual chick in the center of an uncovered opaque plastic container (70 cm diameter × 70 cm height) and covered it with a cloth until it was calm and quiet (<15 s). Afterwards, we uncovered and exposed the chick to a playback of 3 mew calls (18 s in total) followed by 21 s of silence and then another playback of adult alarm calls for 35 s. For all focal chicks, we used the same set of playbacks (mew and alarm calls), which were previously recorded in the study colony, set at a volume as similar as possible to natural sounds. Gull chicks typically respond to adult mew calls (a parental call related to feeding) by increasing begging behavior (chatter calls) and to adult alarm calls by suppressing activity and vocalization and crouching (Tinbergen 1953). The chick behavior during the tests (74 s) was recorded using a digital video camera (Sony Handycam DCR-SX44) held at 1.2 m above the chick on a tripod. The same researcher (S.C.-M.), blind to the treatment, analyzed the videos to count the number of chatter calls (ie begging behavior: Noguera et al. 2010) in response to mew calls until the end of the silent period (39 s). She also recorded the time taken for each chick to crouch as a response to alarm call playback (ie antipredator behavior: Morales et al. 2018; Fig. 2c). All chicks crouched within the duration of alarm playbacks (ie <35 s). After the tests, the chicks were measured (tarsus and weight) and then returned to their nests. Two captured chicks of age 4 d (on 2 d post implantation) were excluded from behavioral tests because, at this age, chicks start to recognize their own parents and territory and may not behave normal in the experimental arena.

### Parental mobbing behavior

On 5 d post implantation (when chicks were 5 to 7 d old), we tested the defensive response (mobbing) of adult birds to an intruder. Mobbing is an important antipredator behavior in which colonial birds collectively harass and attack a predator or any other intruders, including humans, to reduce the immediate risk (Carlson and Griesser 2022). In gulls, mobbing includes circling above, squawking, and direct attacks by diving over the intruder. To record this behavior, we performed a standardized test consisting of 1 or 2 researchers (S.C.-M. and A.V.) positioned over the focal chicks, remaining immobile for 1 min. Mobbing behavior was recorded by a 180° video camera (Apeman A80) mounted on top of a helmet worn by the researcher (S.C.-M.). In all cases, the proximity of the researcher to the chicks triggered the parents’ mobbing behavior. In the videos, blind to the treatment, we recorded the number of dive-bombings and squawking calls. Mobbing behavior was recorded in 55 nests (*N* = 14 corticosterone implanted, *N* = 14 corticosterone neighbor, *N* = 13 placebo implanted, and *N* = 14 placebo neighbor nests).

### Adult behavior in their breeding territory

On 5 d post implantation, immediately after testing mobbing behavior, we recorded the behavior of adults in their breeding territory by placing video cameras (Victure HC200) on wooden posts (1 m high) at a distance of 4 to 5 m from each focal nest. This positioning ensured optimal observation of individuals with high-quality images, while minimizing disturbance to the birds. The cameras were programmed to activate upon detecting movement and record parental behavior for 10 min at a time. We positioned cameras in each individual nest in the morning (10 to 13 h) or the afternoon (14 to 18 h). The observation time, ie the time that the camera remained in each focal nest, was on average 160 ± 3.8 min; the average recording time per nest, ie time that the video was activated and recorded, was 103 ± 6.5 min.

For data analysis, we excluded the nests where adult behavior was not recorded at all or poorly recorded, mainly because adults moved in and out of the camera's field of view. In those excluded nests, recording time was <20% of observation time. We were able to obtain effective video recordings of parental behavior in 44 nests (*N* = 10 corticosterone implanted, *N* = 13 corticosterone neighbor, *N* = 10 placebo implanted, and *N* = 11 placebo neighbor nests). In these videos, we quantified the total number of chick feeding events per observation time (feeding events/observation time). We also quantified the numbers of grooming, aggressive, and resting events of individual adults in each video recording. Grooming was counted when an individual preened their feathers, and resting was counted when its head was resting on the body. Lastly, aggressive behavior was recorded as the number of direct attacks on conspecific intruders. All behaviors, except resting events, directly triggered the video recording. Resting events were observed in 10 min recordings after the camera was activated by movements of chicks or adults as well as vegetation. Although the recording time accounted for >50% of the observation time in all nests, the results regarding resting behavior should be taken with caution, as this behavior might be underestimated. For individual nest estimates of each behavior, we calculated the total number of the behavior displayed by both parents divided by the total video recording time and average number of parents in videos. Chick behavior was not recorded because, in most cases, the small chicks were not visible on the video due to dense vegetation in the nest territory.

### Chick sampling after exposure to exogenous corticosterone

On 8 d post implantation (when chicks were 8 to 10 d old), we captured and blood sampled all focal chicks that we found alive in the experimental nests (*N* = 128). Ten chicks were found dead (3 corticosterone implanted, 1 corticosterone neighbor, 3 placebo implanted, and 3 placebo neighbor chicks) and 21 chicks could not be located. After a second blood sampling (see the “Standardized capture-restraint protocol” section), we measured chicks for tarsus length and marked them with a numbered plastic ring for long-term identification before returning them to their territories.

When chicks were fully grown and near fledging (age 26 to 36 d), we searched for them around the territories. At this stage, the colony is very sensitive to disturbance, so we only sampled those fledglings that were easily located during the visit. Thus, our sampling did not serve to estimate fledging success of the experimental chicks. We blood sampled and measured (tarsus length) all experimental chicks encountered in the focal territories (*N* = 69).

### Standardized capture-restraint protocol

On 8 d post implantation, after the initial blood sampling (for “basal corticosterone levels”), we followed a standardized capture-restrain technique simulating a stressful event to assess stress-induced corticosterone levels (Wingfield et al. 1992; Wingfield 1994). Thus, we kept the chicks in individual cloth bags and suspended them off the ground outside the dense colony. We then obtained a second blood sample from the brachial vein on the opposite wing of the chick 30 min after capture. Plasma corticosterone levels typically increase after stress exposure, peaking within 30 to 60 min. Therefore, we used the second blood sample to measure stress-induced corticosterone levels (Wingfield et al. 1992; Canoine et al. 2002). We additionally estimated the maximal increase in plasma corticosterone, which was calculated by subtracting the basal corticosterone level of each chick from its stress-induced corticosterone level.

### Laboratory analyses

We determined chick sex by molecular sexing (Fritlolfsson and Ellegren 1999) using DNA extracted from blood samples of hatchlings with commercial kits (Quick-DNA Miniprep Plus Kit; Zymo Corp).

We quantified corticosterone concentration using a commercial kit (ELISA Kit EIA-4164 from DRG Diagnostics, Marburg, Germany), following the manufacturer's instructions, by using blood samples collected on the day of implantation (prior to the start of the experiment), 2 d post implantation (in the subsample of implanted chicks, see above), 8 d post implantation (2 bleeding events: baseline and stress induced), and at fledging. Briefly, plasma samples (20 μl) separated from blood were incubated with a corticosterone-horseradish peroxidase conjugate for 60 min in a flat-bottom microtiter plate. The microtiter plate was then washed 3 times and allowed to react with a substrate solution that gave rise to a blue–green complex, of which the absorbance at 450 nm (Synergy 2 Multi-Mode Microplate Reader, Bio-Tek Instruments, Inc.) was inversely proportional to the concentration of corticosterone in the sample. All samples were analyzed in duplicate. In serial dilution of 3 samples, a previous gull study using this commercial kit found that the optical density of the dilution curve paralleled the standard curve and that the expected and observed concentration in the diluted samples followed the expected linearity (Cortés-Manzaneque et al. 2025a). The mean intra-assay and inter-assay coefficients of variation were 8.8% and 10.6%, respectively.

### Statistical analyses

We performed all data analyses using R version 4.1.3 (R Core Team 2022). Before analysis, we transformed some variables (logarithm [log], square root [sqrt], or ordered quantile [orq] transformation; see below) to meet model requirements, including homoscedasticity and normality when necessary. We standardized all variables into *z*-scores (mean = 0, standard deviation = 1) and report standardized coefficients (*β*) and 95% confidence intervals (CIs) for all models, excluding nonsignificant interaction terms (Engqvist 2005). Linear mixed model and generalized linear mixed model analyses were performed by using the *glmmTMB* package (Brooks et al. 2017). In the mixed models with singular fits (ie analyses of sex ratio, longitudinal basal corticosterone levels, and feeding, grooming, and resting behaviors), we specified Gamma priors to estimate the variance parameters of the random effects (Chung et al. 2013) by using the function implemented in the *glmmTMB* package (Bolker 2024). We assessed significance by the Wald statistics using the *car* package (Fox and Weisberg 2019). We estimated model-predicted means (adjusted for the effects of other terms in the model) and 95% CIs estimated from 10,000 bootstrapped simulations of the posterior distribution of model parameters (Brooks et al. 2017) using the *parameters* package (Ludecke et al. 2020) for lineal models and the function provided by Santon and colleagues (2023) for mixed models. Partial effects (ie controlling for other predictors in the model) were illustrated using model residuals and considering the predicted effects for the predictor(s) of interest while controlling all other predictors (setting factors to the average effect and covariates to their median values).

#### Sampling bias test

We first tested whether the plasma corticosterone level at 0 d post implantation (before experimental manipulation), egg volume, and sex ratio did not differ between the experimental groups. Linear mixed models were used for the analyses of corticosterone and egg volume, and a generalized linear mixed model with a binomial distribution for sex ratio analysis. All these models included the nest dyad treatment (corticosterone and placebo), brood manipulation (implanted and neighbor), and their interaction as fixed effects, and brood identity nested within nest dyad identity as random effects.

#### Immediate effects of exogenous corticosterone on the implanted chicks

In a subset of implanted chicks (*N* = 38 chicks; 1 chick per implanted brood, see above), we evaluated the effects of the exogenous corticosterone exposure on basal corticosterone level and behaviors (antipredator behavior and chatter calls) at 2 d post implantation by 2 linear model analyses and a generalized linear model analysis. In the first linear model for basal corticosterone at 2 d post implantation, implant type (corticosterone and placebo), chick sex, age (days), and the corticosterone level at 0 d post implantation were included as independent variables. In this analysis, corticosterone level at 0 d post implantation (ie before the experimental manipulation) was included as a covariate because it reflects genetic and/or maternal effects, which may persistently influence the endocrine profiles during early development (Hayward and Wingfield 2004; Kim et al. 2013; Jenkins et al. 2014; Noguera and Velando 2019; Ruiz-Raya et al. 2023).

In the second linear model for antipredator behavior (time to crouch), we tested the effects of implant type, chick sex, and age. The antipredator behavior, ie “time to crouch,” was orq-normalized using the orderNorm transformation implemented in the *bestNormalize* package (Peterson 2021). Ordered quantile normalization ensures optimal normalization by ranking the data and applying an inverse normal transformation, maintaining the relative order of observations while achieving a Gaussian-like distribution (eg original quantiles for “time to crouch”: 0% = 0.000, 25% = 3.730, 50% = 4.660, 75% = 6.035, 100% = 27.000). We analyzed the number of chatter calls in a generalized linear model with a Poisson error distribution, including implant type, chick sex, age, and body mass at 2 d post implantation as independent variables.

#### Long-term effects of exogenous corticosterone on the implanted and neighboring chicks

We tested the effects of nest dyad treatment (corticosterone and placebo), brood manipulation (implant and neighbor), and their interaction on basal corticosterone levels (sqrt-transformed) of all experimental chicks in a linear mixed model analysis, including sampling time (as a 2-level factor, 8 d post implantation and fledging), chick sex, age at implantation (days), and corticosterone level at 0 d post implantation (sqrt-transformed) as fixed effects, and individual, nest dyad, and brood identities as nested random effects. Additionally, we also analyzed basal corticosterone levels separately for each sampling time (ie linear mixed models for 8 d post implantation and for fledging), including age (days) as an additional fixed term, instead of age of implantation.

We analyzed stress-induced corticosterone level (sqrt-transformed), measured by the standardized capture-restraint protocol at 8 d post implantation in a linear mixed model, including nest dyad treatment, brood manipulation, nest dyad treatment × brood manipulation interaction, age at sampling, sex, and initial corticosterone level (sqrt-transformed) as fixed effects, and nest dyad and brood identity as nested random effects. Similar results were obtained when we analyzed the corticosterone maximal increase (see above; results not shown).

We analyzed tarsus length in a linear mixed model, including nest dyad treatment, brood manipulation, nest dyad treatment × brood manipulation interaction, sampling time (8 d post implantation and fledging), and egg volume as fixed effects, and individual, nest dyad, and brood identities as nested random effects. Additionally, we also analyzed tarsus length separately for each sampling time (ie linear mixed model at 8 and the other at fledging).

#### Effects of corticosterone implants in chicks on the behavior of adult birds

We tested the effects of nest dyad treatment, brood manipulation, and their interaction on adult antipredator mobbing behaviors (number of dive-bombings and squawking calls) per nest using 2 generalized linear mixed models with a Poisson error distribution, including the numbers of parents and researchers present during recording sessions (either 1 or 2 individuals in both cases) as additional fixed terms and nest dyad identity as a random factor. Because weather may influence the propensity of gulls to exhibit mobbing defense (Burger and Gochfeld 1983), weather (rainy or sunny) was included as another additional fixed term.

We also analyzed the interacting effects of nest dyad treatment and brood manipulation on parental behaviors video recorded at the nest (frequency of feeding events, sqrt-transformed, sqrt-transformed; grooming, sqrt-transformed; aggression, log-transformed; resting, sqrt-transformed) in 4 linear mixed models, including number of chicks in the brood and time of day (morning or afternoon) as additional fixed effects and nest dyad identity as a random factor. When the interaction between dyad treatment and brood manipulation was significant (aggression behavior, see “Results”), we performed a planned comparison between corticosterone and placebo adults in both neighbor and implanted nests using the “emmeans” package (Lenth 2025).

### Ethics

The study complied with the standards of animal experimentation and animal welfare established under current Spanish law (RD53/2013), and our protocol was reviewed and approved by the Animal Experiment Ethics Committee of the Xunta de Galicia (ES360570181401/22/FUN01/BIOL AN08.AVR01). Permissions for fieldwork were granted by the authorities of Parque Nacional de las Islas Atlánticas and approved by the Xunta de Galicia (CVE: j4VETEBuA8).

## Results

Egg volume, sex ratio, and basal corticosterone level of hatchlings were balanced between samples assigned to different experimental groups (corticosterone implanted, placebo implanted, corticosterone neighbor, and placebo neighbor), as there was no effect of nest dyad treatment and brood manipulation (Table S1).

### Immediate effects of exogenous corticosterone on the implanted chicks

**Fig. 3.**
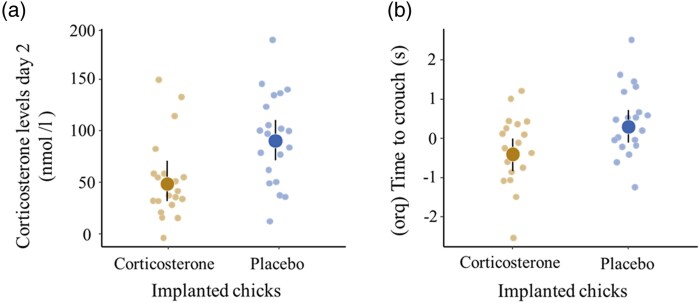
Early effects of the corticosterone implant on a) basal plasma corticosterone levels (*N* = 20 placebo, *N* = 20 corticosterone), and b) antipredator behavior measured as time to crouch in response to adult alarm calls (orq-transformed; *N* = 20 placebo, *N* = 18 corticosterone) in the subset of the implanted chicks at 2 d post implantation. Large dots denote model-predicted means with their 95% CIs, and small dots denote partial effects controlling for other predictors in the model.

In the subset of implanted chicks, which were blood sampled at 2 d post implantation, the corticosterone-implanted chicks showed lower levels of basal corticosterone than the placebo-implanted chicks (*β* [corticosterone] = −0.80, 95% CI: −1.37 to −0.23, *F*_1,34_ = 11.89, *P* = 0.001; Table S2 and Fig. 3a). The corticosterone-implanted chicks crouched significantly faster than the placebo-implanted chicks in response to simulated alarm calls (*β* [corticosterone] = −0.73, 95% CI: −1.34 to −0.11, *F*_1,33_ = 4.86, *P* = 0.034; Table S2 and Fig. 3b). Corticosterone-implanted chicks emitted fewer chatter calls than the placebo-implanted chicks, but this difference was not significant (*β* [corticosterone] = −0.37, 95% CI: −0.87 to 0.13, χ^2^ = 2.14, *P* = 0.143; Table S2).

### Long-term effects of exogenous corticosterone on the implanted and neighboring chicks

Basal corticosterone levels of the chicks at 8 d post implantation and fledging did not differ between the nest dyad or brood manipulation groups (Tables 1 and S3). Similar results were obtained when basal corticosterone levels at 8 d post implantation and fledglings were analyzed separately (Table S4). Although the implanted chicks (both corticosterone and placebo) tended to show lower levels of basal corticosterone at 8 d post implantation than did the nonimplanted chicks, this effect was not significant (*β* [nonimplanted] = −0.38, 95% CI: −0.01 to −0.77, χ^2^ = 3.67, *P* = 0.056; Table S4).

**Table 1.** Results of linear mixed models of basal corticosterone levels and tarsus length at 8 d post implantation (*N* = 128) and fledging (*N* = 69) and stress-induced corticosterone level at 8 d post implantation (*N* = 128) in yellow-legged gull chicks.

	Dependent variables								
	Baseline corticosterone			Stress-induced corticosterone			Tarsus length		
Fixed effects	*β*	*χ* ^2^	*P*-value	*β*	*χ* ^2^	*P*-value	*β*	*χ* ^2^	*P*-value
**Intercept**	0.04 (−0.30, 0.38)			−0.38 (−0.75, −0.01)			−0.77 (−0.85, −0.68)		
**Nest dyad treatment (corticosterone)**	−0.16 (−0.54, 0.21)	0.76	0.382	0.46 (0.04, 0.88)	4.51	**0.034**	0.02 (−0.07, 0.11)	0.25	0.614
**Brood manipulation (neighbor)**	0.24 (−0.07, 0.55)	2.47	0.116	0.32 (−0.06, 0.70)	2.68	0.101	0.01 (−0.07, 0.08)	0.01	0.932
**Sex (male)**	−0.13 (−0.42, 0.16)	0.81	0.369	−0.02 (−0.33, 0.29)	0.02	0.885	0.09 (0.01, 0.17)	27.60	**<0.001**
**Corticosterone day 0**	0.03 (−0.12, 0.18)	0.16	0.684	0.10 (−0.07, 0.27)	1.25	0.263	…		
**Sampling time (fledging)**	−0.04 (−0.30, 0.22)	0.11	0.740	…			1.89 (1.81, 1.97)	4,848.65	**<0.001**
**Age at implantation or sampling^a^**	−0.07 (−0.22, 0.08)	0.91	0.338	−0.13 (−0.32, 0.07)	1.70	0.193	0.06 (0.02, 0.09)	10.32	**0.001**
**Sampling time × sex**	…			…			0.26 (0.15, 0.37)	20.67	**<0.001**

Random effects	Variance	Variance	Variance
**Individual identity**	0.292	…	0.429
**Nest dyad identity**	0.632	0.663	0.952
**Brood identity**	0.607	1.537	1.282
**Residual**	1.744	1.732	2.284

The effects of nest dyad treatment, brood manipulation, sampling time (2-level factor: 8 d post implantation and fledging), sex, and initial corticosterone level were explored. Significant effects are shown in bold. Nonsignificant nest dyad treatment × brood manipulation interaction was removed from the models.

^a^Age at implantation in the longitudinal analyses of basal corticosterone level and tarsus length, or age at sampling in the analysis of stress-induced corticosterone.

**Fig. 4.**
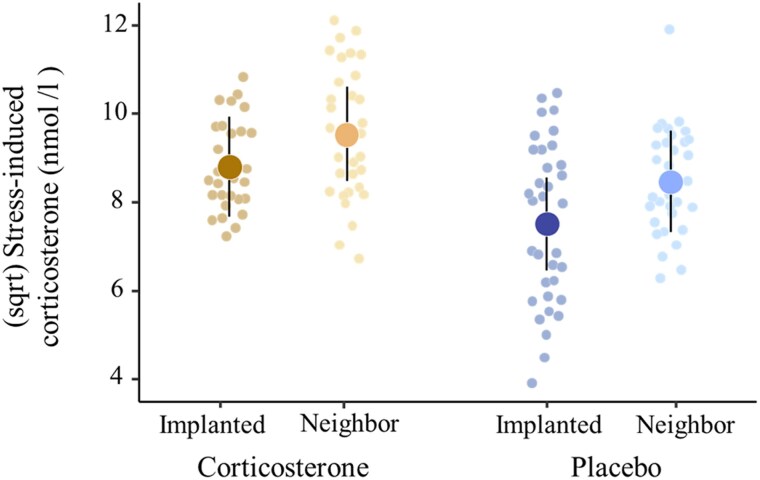
Effects of the paired treatment and brood manipulation on the stress-induced corticosterone levels on day 8 (*N* = 29 corticosterone implanted, *N* = 32 corticosterone neighbor *N* = 34 placebo implanted, and *N* = 33 placebo neighbor chicks). Large dots denote model-predicted means with their 95% CIs, and small dots denote partial effects controlling for other predictors in the model.

Both the corticosterone-implanted chicks and their neighbor chicks showed stronger corticosterone responses than the placebo-implanted chicks and the neighbors during the restraint assays (Nest dyad treatment, *β* [corticosterone] = 0.46, 95% CI: 0.04 to 0.88, χ^2^ = 4.51, *P* = 0.034; Table 1 and Fig. 4). Accordingly, the interaction between nest dyad and brood manipulation was not significant (Table S3).

Tarsus length at 8 d post implantation and fledging did not differ between the experimental groups (Tables 1 and S3). As predicted, males grew faster than females, and the sexual difference was stronger at fledging than at 8 d post implantation (Tables 1 and S4).

### Effects of corticosterone implants in chicks on the behavior of adult birds

**Fig. 5.**
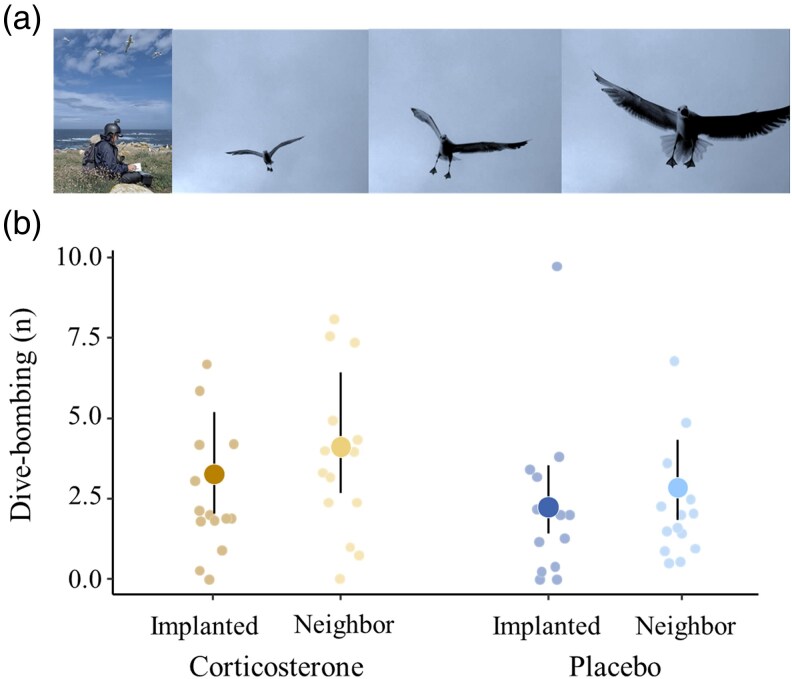
a) Photographs show the researcher with a helmet camera and a sequence of a dive-bombing recorded using the 180° camera. b) Effect of the nest dyad treatment on adult defense behavior, dive-bombing (*N* = 14 corticosterone-implanted nests; *N* = 14 corticosterone neighbor; *N* = 13 placebo implanted; *N* = 14 placebo neighbor). Large dots denote model-predicted means with their 95% CIs, and small dots denote partial effects for each nest, controlling for other predictors in the model.

At 5 d post implantation, the parent birds in the corticosterone dyad nests showed higher numbers of dive-bombings as a response to the standardized (researcher) intrusion than did those in the placebo dyads (nest dyad treatment, *β* [corticosterone] = 0.37, 95% CI: 0.01 to 0.74, χ^2^ = 4.01, *P* = 0.045; Table 1 and Fig. 4), irrespective of the brood manipulation (implanted or neighbor; Tables 2 and S5 and Fig. 5). The number of squawking calls was not affected by the nest dyad treatment, brood manipulation, or their interaction (Tables 2 and S5). The birds showed increased dive-bombing behaviors and squawking calls when the intrusion was made by a single researcher than when 2 researchers visited the nest, and when both parents were present than when only 1 was present (Table 2).

**Table 2.** Results of generalized linear mixed models, with a Poisson error distribution, of adult mobbing behavior (numbers of dive-bombing and squawking calls) in yellow-legged gull nests on day 5 (*N* = 55).

	Dependent variable					
	Dive-bombing			Squawking calls		
Fixed effects	*β*	*χ* ^2^	*P*-value	*β*	*χ* ^2^	*P*-value
**Intercept**	0.90 (0.39, 1.41)			0.06 (−1.00, 1.12)		
**Nest dyad treatment (corticosterone)**	0.37 (0.01, 0.74)	4.01	**0.045**	0.33 (−0.58, 1.23)	0.51	0.476
**Brood manipulation (neighbor)**	0.24 (−0.04, 0.51)	2.80	0.094	0.18 (−0.17, 0.53)	0.99	0.319
**Weather (sunny)**	−0.25 (−0.66, 0.16)	0.23	0.230	−0.22 (−0.98, 0.54)	0.31	0.576
**Researchers**	−0.49 (−0.72, −0.26)	17.33	**<0.001**	−0.68 (−1.21, −0.16)	6.49	**0.011**
**Parents**	0.52 (0.36, 0.69)	37.95	**<0.001**	0.41 (0.19, 0.64)	13.11	**<0.001**

Random effects	Variance	Variance
**Nest dyad identity**	0.274	1.01

Effects of nest dyad treatment and brood manipulation, weather, number of parents, and researchers were explored. Significant effects are shown in bold. Nonsignificant dyad treatment × brood manipulation interactions were removed from the presented models.

**Fig. 6.**
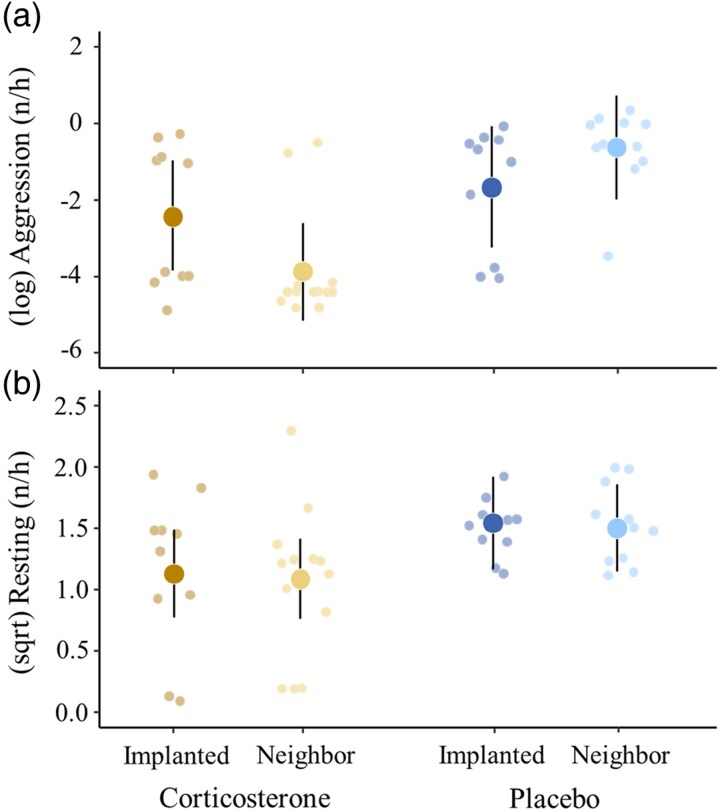
Effects of the nest dyad treatment and brood manipulation on adult behaviors at the focal nest sites, a) aggression (log-transformed) measured as the number of direct attacks on intruders, and b) resting behavior (sqrt-transformed) recorded as the number of head resting behaviors (*N* = 10 corticosterone implanted, *N* = 13 corticosterone neighbor, *N* = 10 placebo implanted, and *N* = 11 placebo neighbor nests). Large dots denote model-predicted means with their 95% CIs, and small dots denote partial effects for each nest controlling for other predictors in the model.

At 5 d post implantation, numbers of chick feeding and grooming did not differ between the experimental groups (Tables 3 and S6). There was a significant interaction effect of the nest dyad treatment and brood manipulation on aggressive behavior (Table 3 and Fig. 6a). Adults in the corticosterone dyads showed a reduced level of aggression compared with those in the placebo dyads, and the difference was more prominent in the neighbor nests than in the implanted nests (pairwise comparisons: neighbor nests: *t*_36_ = 3.98, *P* < 0.001; implanted nests: *t*_36_ = 0.84, *P* = 0.414). Parents of the corticosterone-implanted broods and their neighbors showed a reduced level of resting compared with the adults in the placebo dyads (Fig. 6b and Table 3). Resting behavior was not affected by the brood manipulation or their interaction with the nest dyad treatment (Tables 3 and S6).

**Table 3.** Results from linear mixed models of adult behaviors (feeding, flight, grooming, aggressive behavior, and resting) in yellow-legged gull nests at 5 d post implantation (*N* = 44).

	Dependent variable											
	Feeding			Grooming			Aggressive			Resting		
Fixed effects	*β*	*χ* ^2^	*P*-value	*β*	*χ* ^2^	*P*-value	*β*	*χ* ^2^	*P*-value	*β*	*χ* ^2^	*P*-value
**Intercept**	0.01 (−0.51, 0.54)			0.12 (−0.41, 0.65)			0.21 (−0.32, 0.75)			0.42 (−0.09, 0.92)		
**Nest dyad treatment (corticosterone)**	−0.24 (−0.83, 0.36)	0.56	0.453	−0.47 (−1.09, 0.14)	2.31	0.129	−0.32 (−1.07, 0.43)	10.99	**<0.001**	−0.70 (−1.28, −0.11)	5.40	**0.020**
**Brood manipulation (neighbor)**	0.19 (−0.37, 0.75)	0.44	0.507	0.22 (−0.34, 0.78)	0.59	0.443	0.44 (−0.24, 0.11)	0.19	0.663	−0.08 (−0.63, 0.47)	0.07	0.798
**Time day (afternoon)**	−0.09 (−0.70, 0.53)	0.08	0.773	0.13 (−0.50, 0.76)	0.14	0704	0.01 (−0.27, 0.27)	0.01	0.984	−0.11 (−0.72, 0.50)	0.15	0.695
**Number chicks**	0.27 (−0.02, 0.57)	3.22	0.073	−0.05 (−0.34, 0.25)	0.08	0.784	−0.09 (−0.35, 0.18)	0.41	0.523	0.20 (−1.08, 0.49)	1.96	0.162
**Nest dyad treatment × brood manipulation^a^**	…			…			−1.03 (−1.94, −0.12)	4.88	**0.027**	…		

Random effects	Variance	Variance	Variance	Variance
**Nest dyad identity**	0.156	0.237	1.059	0.211
**Residual**	0.401	0.477	1.673	0.507

Effects of the dyad treatment and brood manipulation, number of chicks in the brood, and time of day were explored. Significant effects are shown in bold.

^a^Nonsignificant interactions were removed from the models.

## Discussion

Our results reveal that corticosterone-implanted gull chicks can trigger stress-related responses in their parents and nearby neighbors, both chicks and adults. The corticosterone-implanted chicks showed lower basal corticosterone levels and enhanced antipredator behaviors than the placebo-implanted chicks only 2 d after the implant. Afterwards, basal corticosterone levels in the growing chicks (at 8 d post implantation) and the grown-up fledglings showed no difference between the experimental groups. However, the corticosterone-implanted chicks and their neighbor chicks at 8 d post implantation showed a more acute stress response to handling than did the placebo implant and their neighbor chicks. Adult birds in the corticosterone-implanted nests and neighbor nests also showed an increased level of mobbing behavior to intruders (researchers) but reduced levels of resting and aggression to conspecifics than did the adults in the placebo dyads. Our results suggest that behavioral responses of stressed chicks can induce similar responses in other social members not directly exposed to the stressor, leading to stress crossover at the colony.

Contrary to our predictions, we found that, 2 d after implantation, the corticosterone-implanted chicks had reduced circulating corticosterone levels compared with the placebo-implanted chicks. In birds, corticosterone implants typically increase basal levels of corticosterone in plasma, but its decrease after implantation has also been reported in several studies (Romero et al. 2005; Busch et al. 2008; Müller et al. 2009; Torres-Medina et al. 2018). This decrease has been interpreted as caused by an overactivation of negative feedback in the hypothalamic–pituitary–adrenal axis of the corticosterone-treated animals, which leads to a low release of endogenous corticosterone from the adrenal glands in stressed animals (Dallman et al. 1992). Because our dose was relatively mild (for comparison, see Noguera et al. 2017; 3.5 μg in a silastic tube), it may have triggered this negative feedback, thereby reducing adrenocorticotropic hormone, adrenal activity, and corticosterone production (Goutte et al. 2011).

In a previous study on the same study population (Noguera et al. 2017), a high dose of corticosterone was implanted in chicks of the same age, and this resulted in elevated basal corticosterone levels above their natural range and impaired growth. The contrasting effects of implantation on corticosterone levels and growth between the previous and present studies may be due to the differences in dose as well as hormone diffusion. Compared with the abrupt release of silastic implants, TRP implants (this study) produce a more gradual release (Quispe et al. 2015), and previous studies in rodents and birds have shown that the continuous exposure to exogenous corticosterone can overload the hypothalamic–pituitary–adrenal axis system, resulting in temporary suppression of endogenous corticosterone (Akana et al. 1992; Vandenborne et al. 2005). Activation of the hypothalamic–pituitary–adrenal axis, coupled with effective negative feedback, may promote stress coping strategies (Zimmer et al. 2019). In our study, the chicks implanted with corticosterone showed a faster crouching response to alarm calls and tended to reduce chatter calls, although the latter effect was statistically not significant. These results are in accordance with the previous study mentioned above (Noguera et al. 2017). Thus, despite the contrasting effects on basal corticosterone, in both studies, the exposure of chicks to exogenous corticosterone induced stress-related behaviors, which are likely to help them to cope with a stressor (eg predation risk) and potentially increase short-term survival (Boonstra 2013).

The effects of corticosterone implants on basal corticosterone disappeared 8 d after implantation in accordance with the expected action of TRP treatment (1 wk; see Müller et al. 2009). However, when the chicks were exposed to a standardized handling stress, the corticosterone-implanted chicks showed a stronger corticosterone release response than did the nonimplanted chicks. Moreover, the same pattern of stress response appeared in their neighboring broods. Previous studies have also found long-lasting effects of TRP treatment on corticosterone response to handling stress, but contrary to our result, the main trend was downregulation of plasma corticosterone (reviewed in Torres-Medina et al. 2018; but see Fairhurst et al. 2013). Although future studies should explain this discrepancy among different studies, the increase in stress response found in our study suggests that corticosterone exposure could lead to long-lasting physiological changes, possibly through epigenetic modifications or changes in sensitivity of the hypothalamic–pituitary–adrenal axis (Romero and Wingfield 2015). Importantly, our results also suggest a crossover effect, where exogenous corticosterone exposure affects not only the stress responses of implanted nestlings but also their nonimplanted neighbor chicks.

The transmission of stress was also evidenced by higher levels of defensive mobbing behavior (dive-bombing attacks) by the parents of the corticosterone-implanted chicks as well as the neighbor adults. During the early chick-rearing period, gull parents interact continuously with their chicks in a variety of contexts (eg prolonged feeding events, protection from sunlight and inclement weather; Tinbergen 1953). Corticosterone-implanted chicks may have transferred stress to their parents (Edgar et al. 2011) through their behavioral responses to stress and other unmeasured changes, eg in their microbiota (Noguera et al. 2018) or odor (Asnani and Ramachandran 1993; Bombail et al. 2018). Familial transmission of stress might help parents to better respond to environmental stressors, such as predators (Boonstra 2013; Brandl et al. 2022). In our study, both parents and chicks in the corticosterone-implanted broods showed improved behavioral strategies (mobbing and crouching, respectively) to cope with predators. Parents of the corticosterone-implanted chicks showed reduced intraspecific aggression. Although the results of resting behavior should be treated with caution, as it was generally underestimated due to the motion-activated video recording method (see “Materials and methods”), the parents of the corticosterone-implanted chicks exhibited reduced resting behaviors in these recordings. Overall, these results suggest behavioral trade-offs between the stress response and other activities. Parents that experienced family-transmitted stress may be more alert to possible stressors (predator cues), thereby reducing the time allocated to other important activities, such as resting or territorial defense (Beauchamp 2015). Decreased intraspecific aggression may also be an adaptive response to facilitate cooperative vigilance and enhance colony defense (Wingfield and Silverin 1986; Krams et al. 2010). Importantly, the neighbor adults and parents of the corticosterone-implanted chicks showed similar behaviors, suggesting that adult behavior can be altered by the transmission of stress beyond the family level.

How did chicks transmit their stress to their neighboring nests? One possibility is that the corticosterone-implanted chicks directly affected the neighbor chicks and adults. Because semiprecocial gull chicks can move around the territory already from age 2 to 3 d, chicks and adults in nearby nests are likely to be exposed to their behavioral and acoustic signals (Beer 1979) and even interact with them (Salas et al. 2024). However, a more plausible explanation is that stress was transmitted indirectly from the stressed parents of the corticosterone-implanted chicks to the neighboring adults, who might then pass it on to their offspring. In our study, we selected dyads of 2 closely located nests with similar laying dates in which frequent social interactions between neighboring adults were expected. In dense gull colonies, territorial adults are highly responsive to calls and behaviors of conspecifics, especially nearby adults (Tinbergen 1953; Yabuta et al. 2010; Shah et al. 2015) with whom they often have agonistic interactions (Burger 1984; Crisologo and Bonter 2017) and display a coordinated group defense strategy against predators (Tinbergen 1953; Kazama and Watanuki 2010). All these social interactions may provide (socially acquired) information about the occurrence of stressful threats (Danchin et al. 2004; Creel et al. 2013). Whatever the mechanism is, our study supports the idea that stressed animals may (directly or indirectly) transfer their stress to social partners and influence how the colony responds to predators.

## Conclusion

Our experimental results show that stressed animals themselves are a source of stress not only for the members of their own family but also for the other close social partners. This transmission of stress may provide short-term benefits by promoting physiological and behavioral responses to cope with stressors, such as antipredator behavior in the presence of predators. However, these responses may also entail costs, eg by reducing the time available for other important activities, such as territory defense, resting, and parental care, thereby affecting reproductive success. The trade-off between fast stress response and its cumulative adverse effects under frequent disturbance (by predators or humans) may shape nest site selection and density in colonial breeding animals. The present study, in accordance with some previous studies (Noguera et al. 2017; Brandl and Farine 2024), suggests that the use of socially acquired information may be a widespread phenomenon in animals and serves as a major driving force in social evolution. Further research is needed to better understand the social and physiological mechanisms underlying the transmission of stress between social partners. Understanding these mechanisms can support management strategies for breeding colonies to minimize stress responses to nonlethal disturbances and reduce stress transmission.

## Supplementary Material

araf085_Supplementary_Data

## Data Availability

Analyses reported in this article can be reproduced using the data provided by Cortés-Manzaneque et al. (2025b).
