# Supplementary material for: Corticosterone-implanted chicks transmit stress to parents and neighbors in a colonial seabird
Source: Behav Ecol. 2025 Jul 28;36(5):araf085. doi: 10.1093/beheco/araf085 (PMC12449063; doi:10.1093/beheco/araf085)
Supplement: araf085_Supplementary_Data [file araf085_supplementary_data.pdf]

SUPPLEMENTARY MATERIAL

Title: Corticosterone-implanted chicks transmit stress to parents and neighbors in a colonial seabird.

Journal: Behavioral Ecology

Authors: Susana Cortés-Manzaneque, Sin-Yeon Kim and Alberto Velando

Grupo Ecoloxía Animal, Centro de Investigación Mariña, Universidade de Vigo, Vigo, Spain

Corresponding author: avelando@uvigo.gal

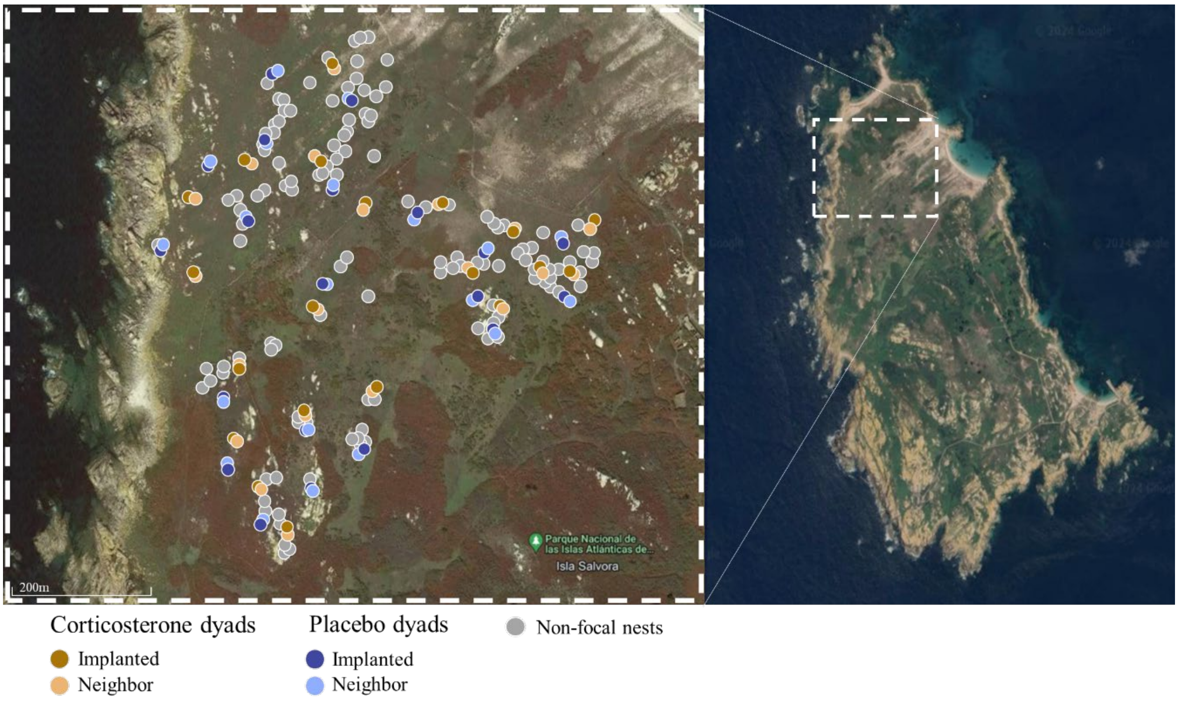

Figure S1. Situation of the 206 marked nests in the breeding colony of the yellow-legged gulls on Sálvora Island. Orange dots indicate corticosterone dyads, blue dots placebo dyads and grey dots indicate non-focal nests used as donors for cross-fostering.

Table S1. Results of full (including non-significant interactions) and final (removing non-significant interactions) linear mixed models (LMMs) of egg volume, sex ratio and initial basal corticosterone levels at 0 day-post-implanation in yellow-legged gull chicks (N = 159) . The effects of nest dyad treatment and brood manipulation were explored.

Full LMM	Dependent variable								
	Egg volume			Sex ratio			Corticosterone levels at day 0		
	β	χ^2	*P*	β	χ^2	*P*	β	χ^2	*P*
Intercept	-0.24 (-0.66, 0.18)			0.01 (-0.73, 0.73)			-0.07 (-0.43, 0.29)		
Nest dyad treatment (corticosterone)	0.38 (-0.22, 0.97)	1.63	0.201	0.11 (-0.92, 1.14)	0.45	0.503	-0.10 (-0.61, 0.41)	1.64	0.200
Brood manipulation (neighbor)	0.19 (-0.38, 0.76)	0.23	0.631	-0.34 (-1.35, 0.67)	0.29	0.593	0.39 (-0.08, 0.85)	2.05	0.153
Nest dyad treatment x Brood manipulation	-0.19 (-0.99, 0.61)	0.21	0.644	0.29 (-1.14, 1.72)	0.16	0.690	-0.30 (-0.96, 0.36)	0.79	0.371
Random effects	Variance			Variance			Variance		
Nest dyad identity	1.819			0.371			19.343		
Brood identity	5.300			0.600			27.889		
Residual	2.863			-			47.033		

Final MM	Dependent variable								
	Egg volume			Sex ratio			Corticosterone levels at day 0		
	β	χ^2	*P*	β	χ^2	*P*	β	χ^2	*P*
Intercept	-0.19 (-0.56, 0.18)			-0.07 (-0.71, 0.57)			0.01 (-0.31, 0.33)		
Nest dyad treatment (corticosterone)	0.28 (-1.16, 0.72)	1.63	0.201	0.26 (-0.49, 1.01)	0.45	0.502	-0.25 (-0.64, 0.14)	1.63	0.200
Brood manipulation (neighbor)	0.10 (-0.30, 0.49)	0.24	0.630	-0.19 (-0.91, 0.52)	0.29	0.592	0.24 (-0.09, 0.57)	2.06	0.151
Random effects	Variance			Variance			Variance		
Nest dyad identity	1.907			0.370			19.530		
Brood identity	5.237			0.601			27.654		
Residual	2.863			-			47.043		

Table S2. Results of linear models of basal corticosterone levels (N = 40) and antipredator behavior (time to crouch) (N = 38) and generalized linear model of begging behavior (number of chattering calls) (N = 38) in yellow-legged gull chicks at 2 day-post-implantation. The effects of implantation treatment, chick sex, initial corticosterone levels and body mass were explored. Significant terms are shown in bold.

	Dependent variable								
	Corticosterone levels day 2			Time to crouch			Chatter call		
	β	$F_{1,34}$	*P*	β	$F_{1,33}$	*P*	β	χ^2	*P*
Intercept	0.41 (-0.12, 0.93)			0.66 (0.09, 1.22)			2.17 (1.75, 2.58)		
Implanted chicks (corticosterone)	-0.80 (-1.37, -0.23)	11.89	**0.001**	-0.73 (-1.35, -0.11)	4.86	**0.034**	-0.37 (-0.87, 0.13)	2.14	0.143
Sex (male)	-0.01 (-0.59, 0.56)	0.054	0.818	-0.59 (-1.22, 0.04)	3.01	0.092	-0.38 (-0.89, 0.13)	2.13	0.145
Chick age	-0.18 (-0.47, 0.17)	1.96	0.171	-0.15 (-0.47, 0.17)	0.95	0.33	-0.11 (-0.38, 0.17)	0.59	0.447
Corticosterone day 0	0.27 (-0.01, 056)	3.75	0.061	-			-		
Body mass[1]	-			-			-0.12 (-0.38, 0.17)	0.82	0.367

[1]Chick body mass was included in the chatter model to control for its known effects on begging intensity (see Methods).

Table S3. Results of full linear mixed models (including non-significant interactions) on basal corticosterone levels and tarsus length growth trajectories from day 8 (N=128) and fledging (N=69); and stress-induced corticosterone levels on day 8 (N=128) in yellow-legged gull chicks. The effects of nest dyad treatment, brood manipulation, sampling time (2-level factor: 8 day-post-implantation and fledging), age at implantation, sex and initial corticosterone levels (on basal corticosterone levels) were explored. Significant terms are shown in bold.

	Baseline corticosterone			Stress-induced corticosterone			Tarsus length		
Fixed effects	β	χ^2	*P*	B	χ^2	*P*	β	χ^2	*P*
Intercept	-0.02 (-0.39, 0.36)			-0.40 (-0.81, 0.01)			-0.77 (-0.86, -0.68)		
Nest dyad treatment (corticosterone)	-0.05 (-0.53, 0.43)	0.75	0.382	0.49 (-0.08, 1.06)	4.50	**0.034**	0.04 (-0.08, 0.15)	0.25	0.614
Brood manipulation (neighbor)	0.35 (-0.07, 0.78)	2.50	0.113	0.35 (-0.17, 0.87)	2.69	0.101	0.02 (-0.09, 0.12)	0.01	0.933
Sex (male)	-0.12 (-0.41, 0.17)	0.69	0.407	-0.02 (-0.33, 0.29)	0.02	0.894	0.09 (0.01, 0.17)	27.73	**<0.001**
Corticosterone day 0	0.02 (-0.13, 0.18)	0.10	0.749	0.10 (-0.08, 0.27)	1.21	0.270	-		
Sampling time (fledging)	-0.04 (-0.30, 0.22)	0.10	0.748	-			1.89 (1.81, 1.97)	4834.16	**<0.001**
Age at implantation or sampling[1]	-0.07 (-0.22, 0.08)	0.97	0.326	-0.13 (-0.33, 0.06)	1.72	0.190	0.06 (0.02, 0.09)	10.28	**0.001**
Nest dyad treatment × Brood manipulation	-0.22 (-0.82, 0.38)	0.56	0.454	-0.07 (-0.81, 0.68)	0.03	0.860	-0.03 (-0.18, 0.12)	0.12	0.730
Sampling time × sex	-			-			0.26 (0.15, 0.37)	20.73	**<0.001**
Random effects	Variance			Variance			Variance		
Individual identity	0.292			-			0.401		
Nest dyad identity	0.637			0.401			0.960		
Brood identity	0.590			1.624			1.277		
Residual	1.744			1.740			2.869		

[1] Age of implantation in longitudinal models (basal corticosterone levels and tarsus length), or age at sampling in the stress-induced corticosterone model

Table S4. Results of additional, separate linear mixed models for each sampling time (8 day-post-implantation and fledging) of basal corticosterone levels and tarsus length. The effects of nest dyad treatment, brood manipulation, sex, age and initial corticosterone levels (on basal corticosterone levels) were explored. Significant terms are shown in bold. Non-significant nest dyad treatment × brood manipulation interactions were removed from the models.

	Dependent variable											
	Baseline corticosterone						Tarsus length					
	8 day-post-implantation			fledging			8 day-post-implantation			fledging		
Fixed effects	β	χ^2	*P*	β	χ^2	*P*	β	χ^2	*P*	β	χ^2	*P*
Intercept	-0.11 (-0.48, 0.25)			0.41 (-0.05, 0.88)			-0.13 (-0.47, 0.21)			-0.56 (-0.98, -0.15)		
Nest dyad treatment (corticosterone)	0.05 (-0.35, 0.45)	0.06	0.812	-0.39 (-0.88, 0.09)	2.43	0.114	-0.01 (-0.41, 0.38)	0.05	0.942	-0.08 (-0.53, 0.37)	0.13	0.724
Brood manipulation (neighbor)	0.38 (-0.01, 0.77)	3.67	0.056	-0.03 (-0.52, 0.46)	0.01	0.907	0.01 (-0.33, 0.33)	0.00	0.995	-0.01 (0.36, 0.35)	0.01	0.985
Sex (male)	-0.22 (-0.54, 0.10)	1.79	0.181	-0.26 (-0.67, 0.14)	1.16	0.202	0.29 (-0.01, 0.60)	3.51	0.061	1.26 (0.91, 1.61)	49.96	**<0.001**
Age	-0.05 (-0.24, 0.13)	0.32	0.571	-0.17 (-0.41, 0.08)	1.18	0.179	0.42 (0.24, 0.60)	20.81	**<0.001**	-0.21 (-0.44, -0.02)	3.14	0.076
Corticosterone day 0	0.15 (-0.02, 0.32)	3.17	0.075	-0.24 (-0.45, -0.03)	5.13	**0.023**	-			-		
Random effects	Variance			Variance			Variance			Variance		
Nest dyad identity	0.001			0.001			0.928			0.439		
Brood identity	1.243			1.275			1.400			0.286		
Residual	1.500			1.247			2.242			0.561		

Table S5. Results of full generalized linear mixed models (including non-significant interactions) with a Poisson error distribution of adult mobbing behavior (number of dive-bombing and squawking calls) in yellow-legged gull nests on day 5 (N=55). Effects of nest dyad treatment and brood manipulation, weather conditions, number of parents and researchers were explored. Significant terms are shown in bold.

	Dependent variable					
	Dive - bombing			Squawking calls		
Fixed effects	β	χ^2	*P*	β	χ^2	*P*
Intercept	0.99 (0.46, 1.52)			0.08 (-1.00, 1.15)		
Nest dyad treatment (corticosterone)	0.21 (-0.28, 0.70)	4.23	**0.040**	0.23 (-1.00, 1.15)	0.50	0.480
Brood manipulation (neighbor)	0.07 (-0.39, 0.52)	2.78	0.095	0.08 (-0.47, 0.63)	0.98	0.322
Weather (sunny)	-0.23 (-0.63, 0.17)	1.29	0.256	-0.17 (-0.97, 0.63)	0.16	0.685
Researchers	-0.49 (-0.71, -0.26)	17.62	**<0.001**	-0.69 (-1.22, -0.16)	6.49	**0.011**
Parents	0.53 (0.36, 0.69)	38.59	**<0.001**	0.39 (0.16, 0.63)	10.59	**0.001**
Nest dyad treatment x Brood manipulation	0.27 (-0.30, 0.84)	0.864	0.353	0.17 (-0.57, 0.92)	0.21	0.646
Random effects	Variance			Variance		
Nest dyad identity	0.250			1.02		

Table S6. Results of full linear mixed models (including non-significant interactions) of adult brood behavior (feeding, flight, grooming, aggressive and resting events) in yellow-legged gull nests on day 5 (N=44). Effects of nest dyad treatment and brood manipulation, number of chicks in the brood and time of day were explored. Significant terms are shown in bold.

	Dependent variable								
	Feeding			Grooming			Resting		
Fixed effects	β	χ^2	*P*	β	χ^2	*P*	β	χ^2	*P*
Intercept	-0.09 (-0.71, 0.53)			0.13 (-0.49, 0.76)			0.35 (-0.25, 0.94)		
Nest dyad treatment (corticosterone)	-0.04 (-0.92, 0.83)	0.56	0.453	-0.50 (-1.40, 0.39)	2.30	0.129	-0.56 (-1.41, 0.28)	5.44	**0.020**
Brood manipulation (neighbor)	0.37 (-0.45, 1.19)	0.45	0.504	0.19 (-0.63, 1.02)	0.59	0.442	0.05 (-0.75, 0.84)	0.07	0.798
Time day (afternoon)	-0.05 (-0.35, 0.25)	0.11	0.741	0.06 (-0.24, 0.37)	0.15	0.701	-0.06 (-0.35, 0.24)	0.17	0.681
Number chicks	0.26 (-0.04, 0.56)	2.81	0.094	-0.04 (-0.35, 0.26)	0.06	0.8070	0.19 (-0.10, 0.48)	1.73	0.188
Nest dyad treatment × brood manipulation	-0.34 (-1.47, 0.78)	0.40	0.526	0.05 (-1.08, 1.18)	0.02	0.892	-0.23 (-1.23, 0.85)	0.17	0.683
Random effects	Variance			Variance			Variance		
Nest dyad identity	0.163			0.241			0.208		
Residual	0.397			0.475			0.507		
